# Retinal capillary degeneration and blood-retinal barrier disruption in murine models of Alzheimer’s disease

**DOI:** 10.1186/s40478-020-01076-4

**Published:** 2020-11-23

**Authors:** Haoshen Shi, Yosef Koronyo, Dieu-Trang Fuchs, Julia Sheyn, Kolja Wawrowsky, Shouri Lahiri, Keith L. Black, Maya Koronyo-Hamaoui

**Affiliations:** 1 Department of Neurosurgery, Maxine Dunitz Neurosurgical Research Institute, Cedars-Sinai Medical Center, 127 S. San Vicente Blvd., Los Angeles, CA 90048 USA; 2 Department of Medicine, Cedars-Sinai Medical Center, Los Angeles, CA 90048 USA; 3 Department of Neurology, Cedars-Sinai Medical Center, Los Angeles, CA 90048 USA; 4 Department of Biomedical Sciences, Division of Applied Cell Biology and Physiology, Cedars-Sinai Medical Center, Los Angeles, CA 90048 USA

**Keywords:** Neurological disease, Neurodegeneration, Dementia, Ocular pathology, Retinal blood vessels, Tight junction

## Abstract

Extensive effort has been made studying retinal pathology in Alzheimer’s disease (AD) to improve early noninvasive diagnosis and treatment. Particularly relevant are vascular changes, which appear prominent in early brain pathogenesis and could predict cognitive decline. Recently, we identified platelet-derived growth factor receptor beta (PDGFRβ) deficiency and pericyte loss associated with vascular Aβ deposition in the neurosensory retina of mild cognitively impaired (MCI) and AD patients. However, the pathological mechanisms of retinal vascular changes and their possible relationships with vascular amyloidosis, pericyte loss, and blood-retinal barrier (BRB) integrity remain unknown. Here, we evaluated the retinas of transgenic APP_SWE_/PS1_ΔE9_ mouse models of AD (ADtg mice) and wild-type mice at different ages for capillary degeneration, PDGFRβ expression, vascular amyloidosis, permeability and inner BRB tight-junction molecules. Using a retinal vascular isolation technique followed by periodic acid-Schiff or immunofluorescent staining, we discovered significant retinal capillary degeneration in ADtg mice compared to age- and sex-matched wild-type mice (*P *< 0.0001). This small vessel degeneration reached significance in 8-month-old mice (*P* = 0.0035), with males more susceptible than females. Degeneration of retinal capillaries also progressively increased with age in healthy mice (*P* = 0.0145); however, the phenomenon was significantly worse during AD-like progression (*P* = 0.0001). A substantial vascular PDGFRβ deficiency (~ 50% reduction, *P* = 0.0017) along with prominent vascular Aβ deposition was further detected in the retina of ADtg mice, which inversely correlated with the extent of degenerated capillaries (Pearson’s *r* = − 0.8, *P* = 0.0016). Importantly, tight-junction alterations such as claudin-1 downregulation and increased BRB permeability, demonstrated in vivo by retinal fluorescein imaging and ex vivo following injection of FITC-dextran (2000 kD) and Texas Red-dextran (3 kD), were found in ADtg mice. Overall, the identification of age- and Alzheimer’s-dependent retinal capillary degeneration and compromised BRB integrity starting at early disease stages in ADtg mice could contribute to the development of novel targets for AD diagnosis and therapy.

**Electronic supplementary material:**

The online version of this article (10.1186/s40478-020-01076-4) contains supplementary material, which is available to authorized users.

## Introduction

Alzheimer’s disease (AD) is a chronic neurodegenerative disease and the leading cause of senile dementia, accounting for 60–80% of all dementia cases [8], in the United States [3]. Preliminary investigations of vascular complications in demented patients revealed atherosclerosis in cerebral blood vessels [5]. To date, a wide spectrum of cerebral blood vessel-related abnormalities have been described as either vascular complications or significant risk factors for AD [53]. Prominent Alzheimer’s cerebral vascular pathologies include vascular perfusion deficits [16, 18, 61], small blood vessel distortions [15, 43, 59, 69], angiogenesis [17, 34], blood–brain barrier (BBB) breakdown [106, 111, 118, 127, 128, 143], neurovascular unit (NVU) degeneration [26, 60, 132], vascular tau accumulation [23, 135], and cerebral amyloid angiopathy (CAA) [7, 40, 133]. The latter is thought to occur in over 85% of AD patients [133]. Moreover, for over two decades, AD-related cerebral pathology has been shown to extend beyond the brain into the retina [33, 37, 44, 58, 77, 78, 81, 125], a central nervous system (CNS) organ that directly connects to and shares many structural and functional features with the brain [41]. In particular, retinal blood vessels in patients with AD are subject to various morphological abnormalities, including disturbed blood flow dynamics, pericyte loss, and amyloid beta-protein (Aβ) deposition [1, 13, 19, 28, 44, 45, 98, 115].

The retina is recognized as the only CNS tissue that can be visually examined noninvasively. As such, retinal imaging may eventually offer a means for implementing early AD screening in large populations. To this end, research investigating Alzheimer’s manifestation in the retina has made considerable progress including demonstrating comprehensive functional decline [50, 87, 101, 107, 119] and pathological damage [1, 4, 13, 19, 28, 33, 37, 44, 45, 58, 77–79, 81, 98, 108, 115, 125, 126]. Optical coherence tomography (OCT) studies identified thinning of the retinal nerve fiber layer in these patients [74, 99, 142], indicating neurodegeneration in the AD retina. Subsequently, retinal amyloidosis, tauopathy, retinal vascular damage, and reduced blood flow were further described by both histological examination and noninvasive retinal imaging [1, 13, 19, 28, 33, 37, 44, 45, 54, 58, 77–79, 86, 110, 115]. Although recent developments in brain imaging modalities have significantly improved differentiation between AD-related and non-AD cerebral pathologies [65], they have not provided a solution in the clinical setting for large-scale screening of at-risk populations in the preclinical or prodromal phases. Retinal imaging techniques, including amyloid or autofluorescence (AF) optical imaging, OCT, OCT angiography (OCTA), and retinal hyperspectral imaging, have already shown positive results in potentially addressing this limitation [56, 73, 77–79, 96, 98, 120]. Importantly, the credibility of evaluating dementia by measuring changes in retinal markers has been established by findings from multiple case studies that successfully correlated retinopathy-related neuronal and vascular pathologies with cognitive decline [10, 19, 20, 32, 38, 94, 121].

Despite the recent advances in understanding Alzheimer’s manifestation in the retina, gaps in knowledge and characterization of retinopathy-related biomarkers currently prevent the reliable diagnosis of AD via retinal imaging. Indeed, the brains of AD patients appear to share many pathological pathways with traditional retinal degenerative diseases such as diabetic retinopathy (DR) and age-related macular degeneration [6, 103, 112, 124]. Similar to AD brain pathology, we recently identified an early and progressive deficiency in vascular platelet-derived growth factor receptor beta (PDGFRβ) and increased apoptosis of pericytes along with vascular amyloidosis in postmortem retinas of mild cognitively impaired (MCI) and AD patients [115]. These findings suggest a blood-retina barrier (BRB) breakdown, but the extent of BRB breakdown in the AD retina compared to that found in traditional retinal degenerative diseases—for example, in the non-proliferative stage of DR—remains unknown [30, 48]. It is important to note that methods for measuring BRB leakage have already been established. Such techniques include fundus fluorescein angiography (FFA) and the recently developed OCT-Leakage, which can be used to detect and quantify BRB leakage in the clinical setting [29, 31, 92]. Breakdown of the BBB in AD brains was confirmed by discoveries of leakage of vascular albumin and globulins [106, 111, 143] and by a recent study using MRI to measure BBB permeability and cerebral blood flow (CBF) [128]. Moreover, another MRI study from the same group demonstrated a correlation between global BBB leakage and cognitive decline in patients with early AD [127]. As such, the current study was designed to further evaluate the integrity of BRB in the AD retina by measuring capillary degeneration and cell–cell junction molecules in relation to AD hallmarks, ultimately contributing to the development of retinal imaging techniques for AD detection and diagnosis.

Here, we investigated retinal microvascular degeneration and BRB integrity in the double-transgenic APP_SWE_/PS1_∆E9_ (ADtg) mouse model as compared to the non-tg wild-type (WT) littermates. We assessed retinal vascular pathology at different mouse ages. First, retinal blood vessels from ADtg mice and WT controls were isolated and stained to quantify degenerated capillaries, a hallmark pathological feature of early-stage DR. Next, pericyte loss and vascular Aβ deposition were evaluated and correlated with capillary degeneration. We identified intensely degenerated capillaries, vascular Aβ deposition, and pericyte loss in ADtg mice, so we further measured cell–cell tight junction molecules and retinal vascular leakage to evaluate the integrity of the BRB. Overall, we demonstrated cellular and molecular BRB disruptions that were linked with retinal vascular amyloidosis in ADtg mice.

## Materials and methods

### Mice

Double-transgenic B6.Cg-Tg (APP_SWE_/PS1_∆E9_)85Dbo/Mmjax hemizygous (ADtg) mice (MMRRC stock #34832-JAX|APP/PS1) and their non-tg littermates (WT control non-AD groups) were used for this experiment. All mice had the genetic background of B6. The animals were purchased from MMRRC and later bred and maintained at Cedars-Sinai Medical Center. The mouse experiments were conducted in accordance with Cedars-Sinai Medical Center Institutional Animal Care and Use Committee (IACUC) guidelines under an approved protocol. The first cohort of 51 mice, age- and sex- matched, were divided into several groups: 4-month-old perfused WT (n = 8), 4-month-old perfused ADtg (n = 8), 8-month-old perfused WT (n = 8), 8-month-old perfused ADtg (n = 8), 8-month-old non-perfused ADtg (n = 3), 12-month-old perfused WT (n = 8), and 12-month-old perfused ADtg (n = 8). The animals were deeply anesthetized with ketamine/xylazine (40–50 mg/kg) before being euthanized by either transcardial perfusion (0.9% ice-cold sodium chloride supplemented with 0.5 mM EDTA) or cervical dislocation (non-perfused group). The animals’ eyes were dissected, and the retinas immediately isolated as previously described [115]. Following isolation, the retinas were processed differently for various purposes. For vascular isolation, retinas were fixed in 4% paraformaldehyde (PFA) solution for 7 days before proceeding to isolation and staining; for protein isolation, retinas were immediately sonicated in RIPA lysis buffer (Thermofisher Scientific, #89900) with proteinase inhibitor and phosphatase inhibitor (Thermofisher Scientific, #78440); for retinal cross-section, whole eyes were fixed in 4% PFA for 30 min in PBS, then transferred to 4% PFA containing 30% sucrose for cryoprotection at 4 °C.

A second cohort of mice (n = 10; average age of 6 months old) was used to assess BBB permeability by tracer infusion: 5 WT and 5 ADtg age- and sex- matched mice received 50 µL of Texas Red-dextran 3 kD (0.25%) and fluorescein isothiocyanate (FITC)-dextran 2000 kD (0.25%) via tail vein before being euthanized by perfusion 30 min later (procedure and tissue collection as described above). Retinas were isolated immediately after mice were euthanized, and then were mounted to microscopic slides with ProLong Gold antifade reagent with 4′,6-diamidino-2-phenylindole (DAPI; Invitrogen #P36935). For purposes of quantification, images were obtained using an Axio Imager Z1 fluorescence microscope (Carl Zeiss MicroImaging, Inc.) equipped with ApoTome, AxioCam MRm, and AxioCam HRc cameras (for more details see *Stereological quantification* below).

Another set of mice (n = 8; at the age of 8, 12 and 16 months) underwent non-invasive retinal imaging after intra peritoneal (IP) injection of Fluorescein (2%; 15 µL; Ak-Fluor #17478-253-10) to assess retinal microvascular leakage. Representative live images were taken during ten to thirty minutes post-injection.

### Retinal vascular isolation and immunofluorescence staining

The trypsin-induced retinal digestion and vascular network isolation technique was originally developed in 1993 and subsequently modified by replacing trypsin with commercially available elastase [129]. Our modified protocol has been previously described [115]. Briefly, retinal strips fixed in PFA were first washed in running distilled water overnight, then digested in 40 U/mL elastase solution (Merck Millipore, #324682) for 2 h at 37 °C. After initial digestion, the tissues were incubated in activation solution (Tris buffer at pH 8.5) overnight for extensive digestion. The next day, the retinas were transferred to Superfrost microscope slides with 1 × PBS, then carefully cleaned with a rat whisker tool under a dissecting microscope to remove unwanted tissue. After the nonvascular tissues had been cleaned, 1 × PBS was applied three times to wash the isolated vascular tissues. Samples of isolated retinal vasculature were then mounted differently for immunofluorescence staining or periodic acid-Schiff (PAS) and hematoxylin staining. For immunofluorescence staining, the tissues were mounted on slides carefully without prior dehydration, then incubated in blocking buffer (Dako #X0909) for 1 h at room temperature (RT). Tissues were then incubated overnight at 4 °C with the following primary antibody combinations: 4G8/lectin/PDGFRβ and 11A50-B10/lectin/PDGFRβ; for a complete list of primary and secondary antibodies as well as other labeling compounds used in this study see Table 1. Tissues were then washed three times with PBS and incubated with secondary antibodies for 2 h at RT. Tissues were further washed with PBS three times, and then vascular trees were mounted using ProLong Gold antifade reagent with DAPI (Invitrogen #P36935). For quantification, images were obtained using an Axio Imager Z1 fluorescence microscope (Carl Zeiss MicroImaging, Inc.) equipped with ApoTome, AxioCam MRm, and AxioCam HRc cameras (for more details see *Stereological quantification* below). For representative images, Z-stack images were repeatedly captured at the same tissue thickness by using a Carl Zeiss 780 confocal microscope (Carl Zeiss MicroImaging, Inc.) or a Leica SP 5 WLL confocal microscope (Leica Microsystems). Routine controls were processed using identical protocols while omitting the primary antibody to assess nonspecific labeling.

**Table 1:** List of antibodies and reagents for imaging

Antibodies or reagents	Source species	Dilution	Application	Commercial source	Catalog. #
*Primary antibody*					
PDGFRβ pAb	Goat	1:200	IF	R&D systems	AF385
4G8 mAb	Mouse	1:200	IF	Biolegend	800701
Alexa Fluor 488-conjugated tomato lectin	*Lycopersicon esculentum*	1:200	IF	Dylight	DL-1174
Aβ_40_ (11A50-B10) mAb	Mouse	1:200	IF	Biolegend	805401
CD31 pAb	Rabbit	1:100	IF	Abcam	ab28364
Zonula Occluden-1 pAb	Rabbit	1:50	WB	Thermofisher	61-7300
Claudin-1 pAb	Rabbit	1:75	WB	Thermofisher	51-9000
phospho-NF-κB p65 Ser536 mAb	Rabbit	1:1000	WB	Cell Signaling Technology	3033S
NF-κB p65 mAb	Rabbit	1:1000	WB	Cell Signaling Technology	8242S
β-actin	Mouse	1:1000	WB	Santa-Cruz Biotechnology	Sc-47778
*Secondary antibody*					
Cy3 (anti-rabbit, anti-goat)	Donkey	1:200	IF	Jackson ImmunoResearch Laboratories	
Cy5 (anti-mouse, anti-rabbit)	Donkey	1:200	IF	Jackson ImmunoResearch Laboratories	
Peroxidase Goat Anti-Rabbit IgG (H + L)	Goat	1:10000	WB	Jackson ImmunoResearch Laboratories	
Peroxidase Goat Anti-Mouse IgG (H + L)	Goat	1:10000	WB	Jackson ImmunoResearch Laboratories	
*In vivo retinal live imaging*					
Fluorescein (322.31 Da)	N/A	2%	IVRLI	Dailymed	17478-250-20
*Ex vivo retinal imaging*					
FITC-dextran (2000kD)	N/A	0.25%	EVRI	Sigma Aldrich	FD2000s-5G
Texas Red-dextran (3kD)	N/A	0.25%	EVRI	Thermofisher	D1829
Thioflavin-S	N/A	1%	IF	Sigma-Aldrich	T1892
*Degenerated capillary quantification (PAS)*					
Periodic Acid	N/A	35 mM	DCQ	Milliporesigma	P7875
Schiff	N/A	100%	DCQ	Sigma Aldrich	3952016

*IF* Immunofluorescence, *WB* western blot, *pAb* polyclonal antibody, *mAb* monoclonal antibody, *IVRLI* in vivo retinal live imaging, *EVRI* ex vivo retinal imaging, *DCQ* degenerated capillary quantification, *N/A* not applicable, if not marked otherwise, antibody dilution is indicated for immunofluorescent assay

### Periodic acid-Schiff staining of isolated retinal microvasculature

For PAS staining, samples of isolated retinal microvasculature were mounted differently after elastase digestion and clearing. Specifically, the isolated retinal microvasculature was dried overnight after being mounted on glass Superfrost Plus microscope slides (Fisher Scientific, #12-550-15). On the following day, the samples were first rehydrated in distilled water for 15 min. The rehydrated samples were then incubated with periodic acid (MilliporeSigma, #P7875) solution at a concentration of 35 mM at RT for 8 min, followed by a brief dipping in distilled water. Afterward, the tissues were stained with Schiff (Sigma-Aldrich, #3952016) for 15 min, followed by three separate extensive washes in running distilled water lasting 5 min each time. The tissues were then stained with hematoxylin (Richard-Allan Scientific, #7231) for 2 min, followed by three 5-minute distilled water washes. After staining, the slides were dehydrated in 70%, 85%, 90% and 100% ethanol, and finally xylene, 2 min for each reagent. Following this, the slides were mounted with Permount mounting medium (Fisher Scientific, #SP15-100). For purposes of quantification, the images were obtained using an Axio Imager Z1 fluorescence microscope (Carl Zeiss MicroImaging, Inc.) equipped with ApoTome, AxioCam MRm, and AxioCam HRc cameras (for more details see *Stereological quantification* below).

### Retinal cross-section, fluorescence and immunofluorescent staining

Eyes preserved in 4% PFA with 30% sucrose were first embedded in OCT compounds on dry ice. Then the retinal cross-sections (10 mm thick) were cut using a cryostat machine (Leica Biosystems) and stored at − 80 °C until use. For immunostaining, retinal cross-sections were incubated in blocking buffer (Dako #X0909) for 1 h at RT, followed by incubation with primary antibodies of rabbit anti-mouse CD31 (1:100; Abcam) and mouse anti-human 11A50-B10 (1:200, Biolegend). Sections were then washed three times in PBS and incubated with secondary antibodies (see Table 1 for details) for 2 h at RT. The sections were then briefly washed twice in PBS for 5 min, then incubated in thioflavin-S (1%, Sigma-Aldrich) for 10 min at RT. Finally, sections were washed with 70% ethanol three times followed by PBS, and then mounted using ProLong Glass antifade reagent (Invitrogen #P36980). Images were obtained using an Axio Imager Z1 fluorescence microscope (Carl Zeiss MicroImaging, Inc.) equipped with ApoTome, AxioCam MRm, and AxioCam HRc cameras. Routine controls included staining of non-Tg mouse retina and ADtg mouse sections that were processed using identical protocols while omitting the primary antibody to assess nonspecific labeling.

### Protein extraction and western blot analysis

Sonicated retinal lysates in RIPA buffer were first centrifuged at 13600 rpm at 4 °C. Afterward, the supernatant was transferred to fresh new tubes. Protein concentration was determined by using the BCA kit (Thermofisher, #23227) and following the standard protocol. Equal amounts of total proteins were then separated onto 4–20% Tris–glycine gels (Invitrogen, #XP04205BOX) and transferred to nitrocellulose membranes. Then, after blocking the membranes in TBST (10 mmol/L Tris–HCl buffer, pH 8.0, 150 mmol/L NaCl, and 0.1% Tween 20) with 5% (w/v) bovine serum albumin (BSA) at RT for 60 min, the membranes were incubated overnight at 4 °C with antigen-specific primary antibodies. The following primary antibodies were used: anti-ZO-1 (1:50; Thermofisher, #61-7300), anti-claudin-1 (1:75; Thermofisher, #51-9000), anti-phospho-NF-κB p65 Ser536 (1:1000, Cell Signaling Technology, #3033S), anti-NF-κB p65 (1:1000, Cell Signaling Technology, #8242S), and anti-β-actin (1:1000; Santa Cruz Biotechnology, #sc-47778). The blots were then incubated with species-specific horseradish peroxidase-conjugated secondary antibodies for 2 h at RT. Proteins were visualized by incubation with a chemiluminescence substrate kit (Thermofisher, #34580). Western blot images were collected (iBright imaging system; Thermofisher), and the targeted protein expression was quantified using Image Studio Lite software version 5.2 (LI-COR Biosciences, Lincoln, NE) after normalizing to β-actin. One representative blot is shown for each molecule.

### Stereological quantification

**Fig. 1:**
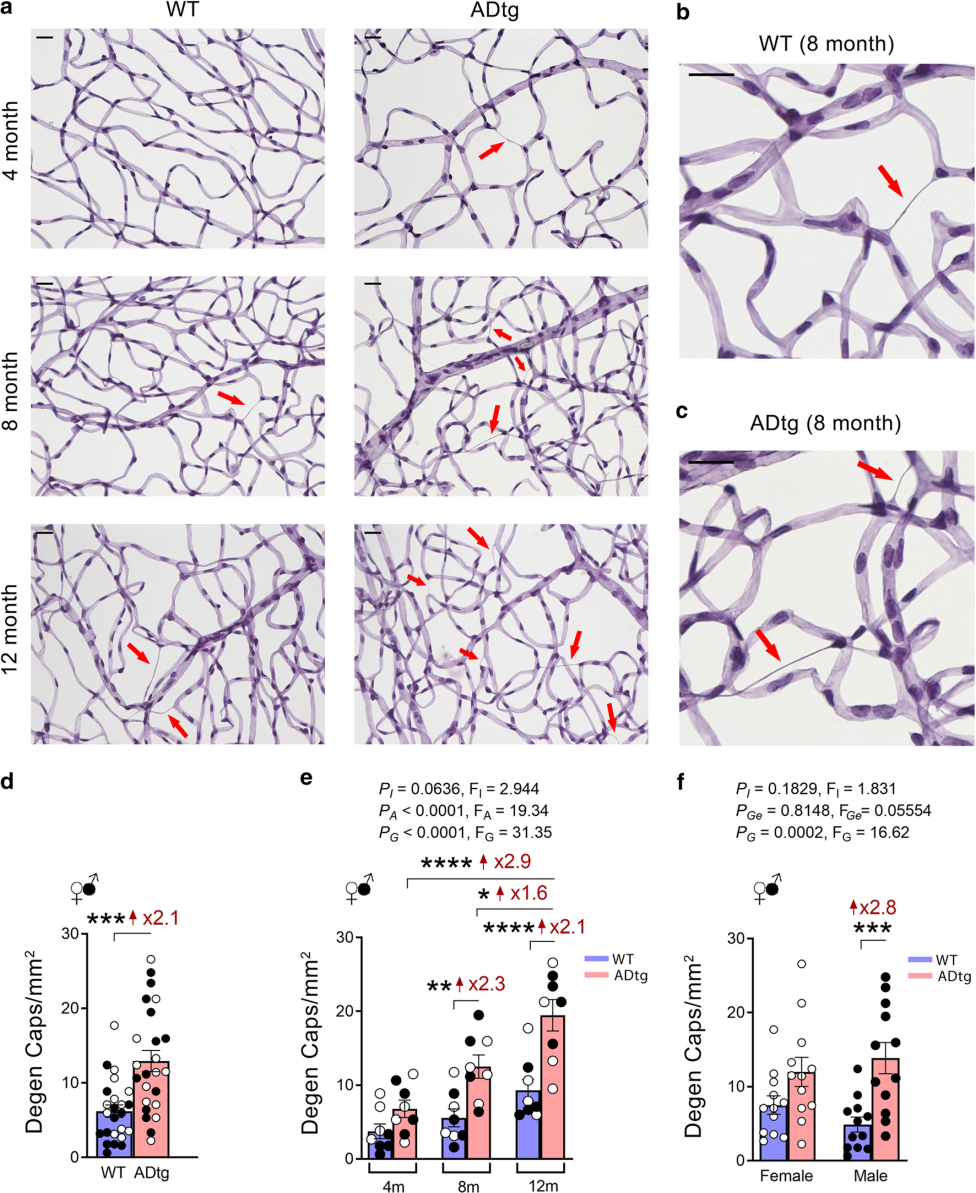
Identification of retinal capillary degeneration in APP_SWE_/PS1_ΔE9_ (ADtg) mice that intensified during disease progression. ADtg mice and their age- and sex-matched wild-type (WT) littermates, males and females in equal number, were used in this study at the age of 4 (n = 16; ADtg = 8 and WT = 8), 8 (n = 16; ADtg = 8 and WT = 8) and 12 (n = 16; ADtg = 8 and WT = 8) months. **a** Representative images of periodic acid-Schiff (PAS)-stained, hematoxylin-counterstained isolated retinal microvasculature from ADtg and matched WT littermates. Acellular degenerated retinal capillaries are indicated by red arrows. **b–c** Higher magnification representative images of acellular, degenerated retinal capillaries from 8-month-old **b**. WT mouse and **c** ADtg mouse (red arrows indicate degenerated capillaries). Scale bars = 20 µm. **d** Count of acellular degenerated retinal capillaries (Degen Caps) in 1 mm^2^ microscopic fields as manually determined in ADtg and WT control mice (mean age: 8 months, 50% females for both WT and ADtg groups). **e–f** Numbers of degenerated retinal capillaries when mice are stratified by mouse genotypes, WT or ADtg, by either **e** age groups of 4, 8 and 12 months or **f** sex. Data from individual mice (circles) as well as group means ± SEMs are shown. Black-filled circles represent males and clear circles represent females. Fold changes are shown in red. *p < 0.05, **p < 0.01, ***p < 0.001, ****p < 0.0001, by two-way ANOVA with Tukey’s post hoc multiple comparison test or by unpaired 2-tailed Student *t*-test. *P* and *F* values of two-way ANOVA refer to comparisons of age groups (*P*_A_, *F*_A_), ADtg versus WT genotype groups (*P*_G_, *F*_G_), gender groups (*P*_Ge_, *F*_Ge_), and overall interactions (*P*_I_, *F*_I_)

Degenerate capillaries were identified as acellular capillary-sized tubes and were manually counted in an average of eight microscopic fields (covering 1.8 × 10^4^-µm^2^ area) per retina (see Fig. 1). Degenerate capillaries were excluded if their average diameter was < 20% the size of surrounding healthy capillaries [80].

**Fig. 2:**
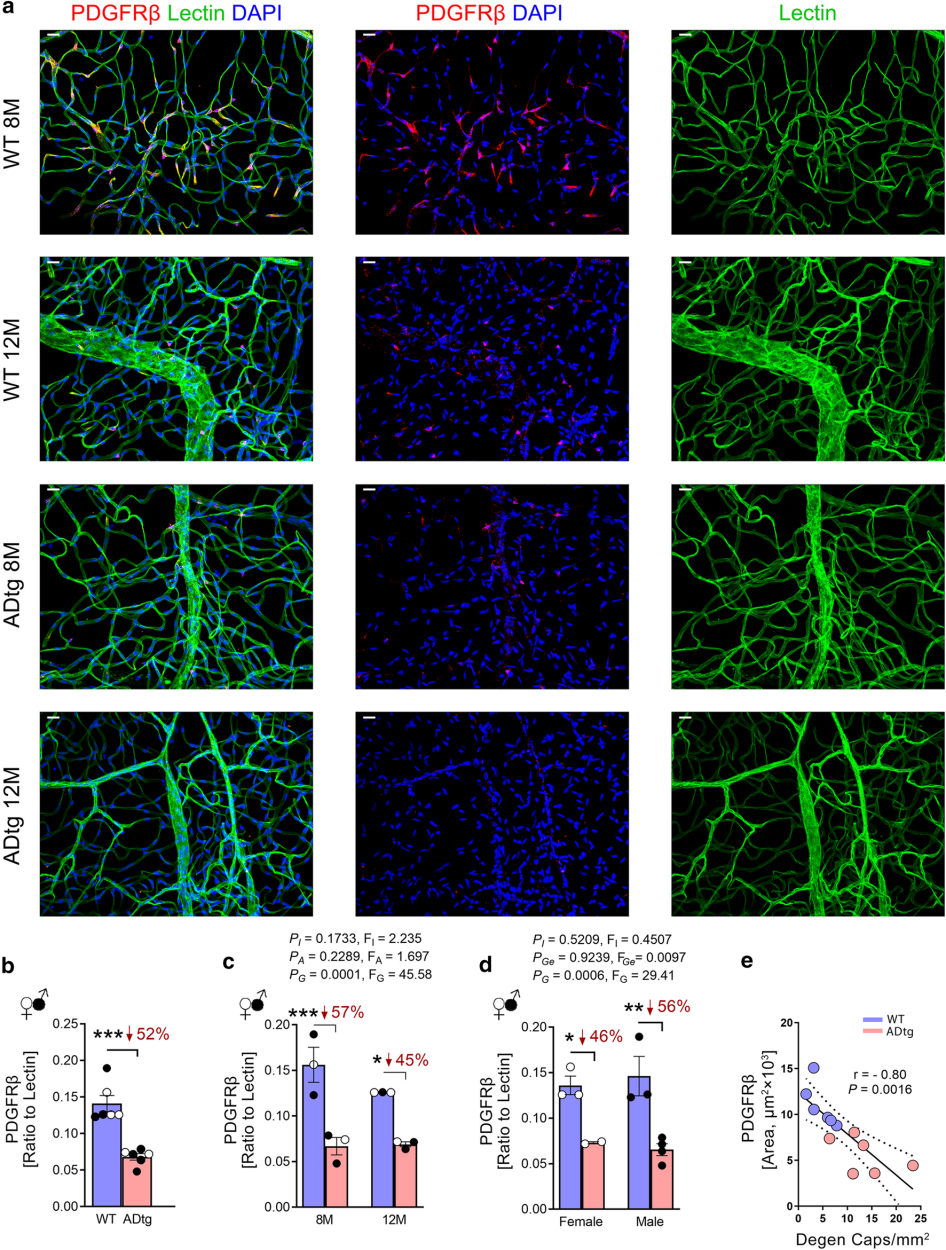
Retinal vascular PDGFRβ deficiency in APP_SWE_/PS1_ΔE9_ (ADtg) mice. A subset of the mouse cohort described in Fig. 1 of age- and sex-matched ADtg and wild type (WT) mice were analyzed at the age of 8 (n = 6; ADtg = 3 and WT = 3) and 12 (n = 6; ADtg = 3 and WT = 3) months. **a**. Representative fluorescence images of isolated retinal microvasculature stained for pericytes (PDGFRβ, red), blood vessels (lectin, green), and nuclei (DAPI, blue) in ADtg and WT littermates. Scale bars = 20 µm. **b**. A quantitative analysis of ratio between PDGFRβ-immunoreactive (IR) area and lectin-IR area in each microscopic field of isolated retinal microvasculature when mice are stratified by genotype of ADtg and WT. **c–d** Ratio of PDGFRβ-IR area to lectin-IR area in each microscopic field in the same mouse cohort when mice are stratified by genotypes of WT vs. ADtg and **c** age of mice by 8 month and 12 month or **d** sex of mice. **e** Pearson’s coefficient (*r*) correlation between retinal PDGFRβ-IR area and degenerated capillary count in the same mice cohort (n = 12). Data from individual mice (circles) as well as group means ± SEMs are shown. Black-filled circles represent males and clear circles represent females; blue-filled circles represent WT mice and pink-filled circles represent ADtg mice. Percentage decreases are shown in red. *p < 0.05, **p < 0.01, ***p < 0.001, by two-way ANOVA with Tukey’s post hoc multiple comparison test. Two group statistical analysis was performed using an unpaired 2-tailed Student *t*-test. *P* and *F* values of two-way ANOVA refer to comparisons of age groups (*P*_A_, *F*_A_), ADtg versus WT genotype groups (*P*_G_, *F*_G_), gender groups (*P*_Ge_, *F*_Ge_), and overall interactions (*P*_I_, *F*_I_)

**Fig. 3:**
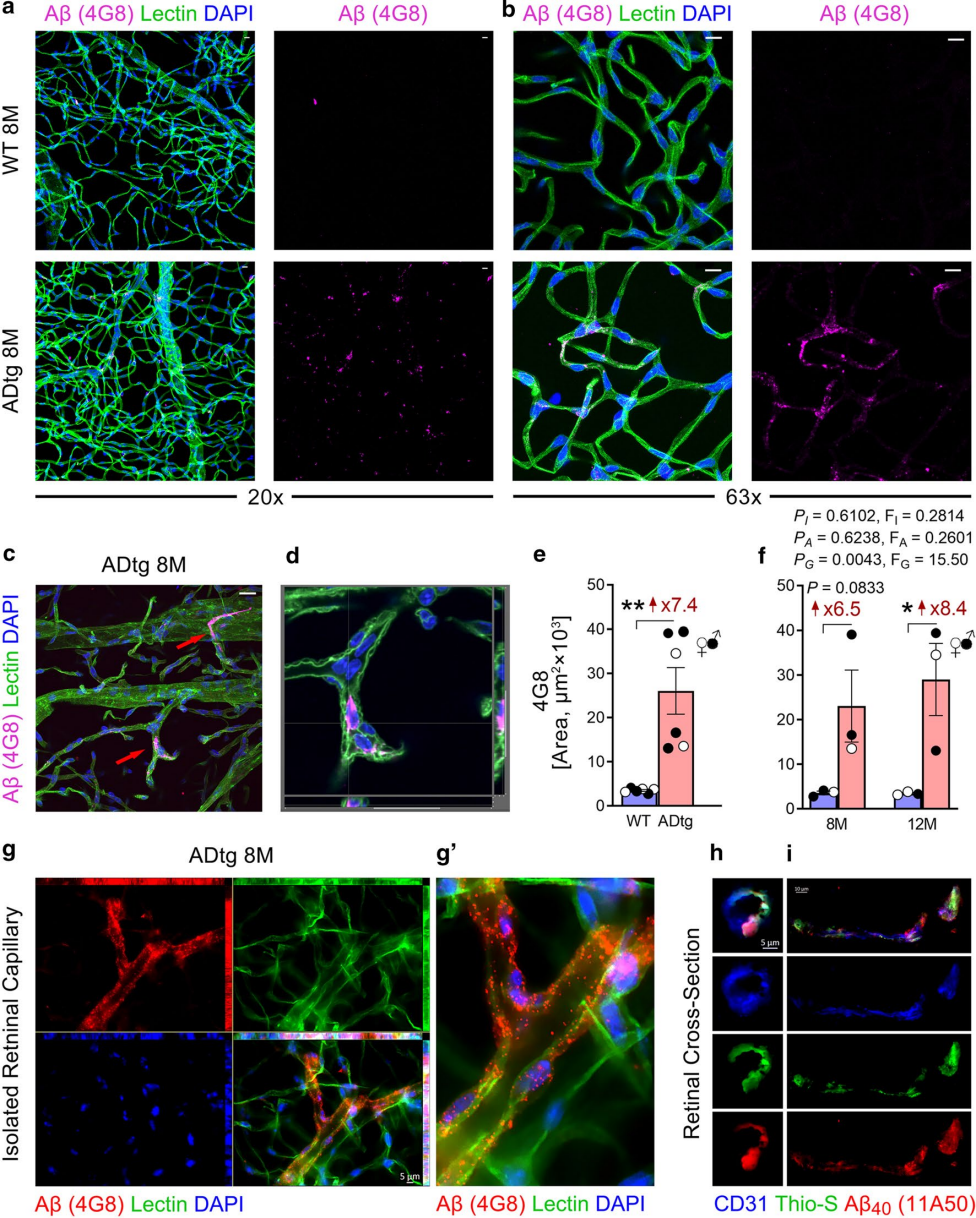
Retinal amyloidosis in blood vessel walls of APP_SWE_/PS1_ΔE9_ (ADtg) mice. **a–b** Representative fluorescence images acquired using **a** 20 × or **b** 63 × microscope objectives, of isolated retinal microvasculature stained for Aβ (4G8, magenta), blood vessels (lectin, green), and nuclei (DAPI, blue) in age- and sex-matched perfused ADtg mice (n = 6) and wild-type littermates (WT; n = 6). Scale bars = 20 µm. **c–d** Representative fluorescence virtual cross-section images acquired using the Leica confocal microscope 63 × objective of the isolated retinal microvasculature stained for Aβ (4G8, magenta), blood vessels (lectin, green) and nuclei (DAPI, blue) in an 8-month-old male ADtg mouse. Scale bar = 20 µm. Arrows indicate vascular Aβ. **e** Quantitative analysis of Aβ (4G8)-immunoreactive (IR) area in each microscopic field of isolated retinal microvasculature from WT vs. ADtg mice. **f** Quantitative analysis of the Aβ (4G8)-IR area stratified by mice age group (8 months vs. 12 months) and genotype (WT vs. ADtg) in the same cohort. **g–g′**. Representative fluorescence images of the isolated retinal microvasculature stained for Aβ (4G8, red), blood vessels (lectin, green) and nuclei (DAPI, blue) in an 8-month-old male ADtg mouse; Aβ signals occur in retinal vessel walls and vascular cells. **h–i**. Representative fluorescence images of fixed retinal cross-section stained for thioflavin-S (Thio-S, green), Aβ_40_ (11A50-B10, red) and blood vessels (CD31, blue) in an 8-month-old male ADtg mouse showing **h** vertical blood vessel and **i** longitudinal blood vessel. Data from individual mice (circles) as well as group means ± SEMs are shown. Black-filled circles represent males and clear circles represent females. Fold changes are shown in red. *p < 0.05, **p < 0.01, by two-way ANOVA with Tukey’s post hoc multiple comparison test or by an unpaired 2-tailed Student *t*-test. *P* and *F* values refer to comparisons of age groups (*P*_A_, *F*_A_), ADtg versus WT genotype groups (*P*_G_, *F*_G_), and interactions (*P*_I_, *F*_I_)

For Figs. 2 and 3, which display isolated retinal blood vessels, quantification was performed using samples from six ADtg mice and six age- and sex-matched littermates. The fluorescence of specific signals was captured using the same setting and exposure time for each image and mouse, with a 10-µm-thick Z-stack by using an Axio Imager Z1 microscope (with motorized Z-drive) with AxioCam MRm monochrome camera version 3.0 (at a resolution of 1388 × 1040 pixels, 6.45 µm × 6.45 µm pixel size, and a dynamic range of > 1:2200, which delivers low-noise images due to a Peltier-cooled sensor). Images were captured at 40× objective, at a respective resolution of 0.25 µm. Fifteen images were obtained randomly from each region of central-, mid-, and far-peripheral retina (five from each region) per subject. Acquired images were converted to gray scale and standardized to baseline using a histogram-based threshold in the NIH ImageJ software program (version 1.52o). For each biomarker, the total area of immunoreactivity was determined using the same threshold percentage from the baseline in ImageJ (with the same percentage threshold setting for all diagnostic groups). The images were then subjected to particle analysis for lectin, Aβ, and PDGFRβ to determine the immunoreactive (IR) area. The ratio of Aβ or PDGFRβ to lectin was calculated by dividing the Aβ-IR or PDGFRβ-IR area by the lectin-IR area in each of the 15 images (described above) and averaging the values per mouse.

For Fig. 5, quantitative analysis of FITC or Texas Red area in each microscopic field of retinal flat-mount was performed based on five ADtg mice and five age- and sex-matched littermates. The fluorescence of specific signals was captured using the same setting and exposure time for each image and mouse by using an Axio Imager Z1 microscope as described above. Images were captured at 40× objective, at a respective resolution of 0.25 µm. Ten images were randomly taken throughout the whole retina, then the images were converted to gray scale and subjected to ImageJ analysis as described above. Particle analyses were performed on FITC and Texas Red signal. Finally, the IR areas were calculated by averaging the ten images per mouse.

### Statistical analysis

GraphPad Prism version 8.3.0 (GraphPad Software) was used for the analyses. A comparison of three or more groups was performed using one-way ANOVA followed by Tukey’s multiple comparison post hoc test of paired groups. Groups with two independent variables/factors were analyzed by using two-way ANOVA followed by Tukey’s multiple comparison test to further understand the interaction between the two independent variables. Two group comparisons were analyzed using a two-tailed unpaired Student *t* test. The statistical association between two or more variables was determined by Pearson’s correlation coefficient (*r*) test (Gaussian-distributed variables; GraphPad Prism). Pearson’s *r* indicates the direction and strength of the linear relationship between two variables. Required sample sizes for two group (differential mean) comparisons were calculated using the nQUERY *t* test model, assuming a two-sided α level of 0.05, 80% power, and unequal variances, with the means and common standard deviations for the different parameters. Results are expressed as means ± standard errors of the means (SEMs). A P value less than 0.05 is considered significant.

## Results

### Age-dependent progressive degeneration of retinal microvessels in healthy and ADtg mice

To explore retinal microvascular degeneration in AD-model mice at ascending ages, we used an elastase-based enzymatic digestion method to isolate the retinal vascular network in 4-, 8- and 12-month-old ADtg mice as compared to their age- and sex-matched non-transgenic littermates. The method was initially developed by Laver et al. [85] and then further modified by Veenstra and colleagues [129]. Specifically, PAS staining was applied to detect polysaccharides, thereby enabling visualization of the vascular network in isolated retinal blood vessels from ADtg mice and WT controls; hematoxylin counterstaining was utilized to highlight the nuclei. We first qualitatively observed more degenerated retinal capillaries in ADtg mice than in the WT control mice, including as early as in 4-month-old mice (Fig. 1a). We determined degenerated retinal microvessels as acellular, basement membrane-only PAS staining-positive structures, which are illustrated at higher magnification microscopic images from mice at the age of 8 months (Fig. 1b–c) and 12 months (Additional file 3: Fig. S1a–b). While both genotypes displayed increasing numbers of degenerated retinal vasculature as the animals grew older, the ADtg mice exhibited an intensified vascular pathology (Fig. 1a–c and Additional file 3: Fig. S1a–b, red arrows).

To quantify retinal vascular degeneration in different mouse genotypes, ages and sex, we manually counted the degenerated retinal microvessels in pre-defined 1 mm^2^ area of each microscopic field (Fig. 1d–f and Additional file 3: Fig. S1c–d). We first analyzed the pooled mice groups to measure the overall AD-associated genotype effect. Regardless of animal age and sex, there was a significant 2.1-fold increase in retinal acellular capillaries in ADtg mice versus WT mice (Fig. 1d). Using two-way ANOVA analysis and Tukey’s post-test, we detected a highly significant increase in amounts of degenerated retinal microvasculature in ADtg mice when compared to WT mice at 12 months of age (Fig. 1e). Furthermore, there was significantly more vascular degeneration in 12-month than in 4-month-old ADtg mice. We also revealed an earlier significant difference in degenerated microvessels between ADtg and WT retinas in mice 8 months of age (Fig. 1e). Although both sexes showed increases in acellular capillaries, only the male mice reached statistical significance between the genotypes (Fig. 1f). Further age comparison analysis per each genotype separately uncovered progressive increases of retinal microvascular degeneration with aging in both the WT and ADtg mice (Additional file 3: Fig. S1c and d, respectively).

### Downregulation of retinal vascular PDGFRβ in ADtg mice

Since pericyte loss generally precedes microvascular degeneration in retinal vascular degenerative diseases [57], we sought to further evaluate whether pericytes undergo degeneration in the retina of ADtg mice. To this end, we immunostained isolated retinal vasculature with PDGFRβ as a pericyte marker in capillaries, together with lectin for blood vessels and DAPI counterstaining for nuclei (Fig. 2a). We found a dramatic loss of PDGFRβ signal in retinal vasculature from ADtg mice compared to WT controls at 8 months of age. A further loss of PDGFRβ expression was evident in older, 12-month-old ADtg mice. Stereological quantification of PDGFRβ immunoreactive (IR) area and PDGFRβ-IR area normalized to lectin-IR area confirmed significant 49% and 52% decreases in vascular PDGFRβ signal, respectively, in ADtg compared to WT control mice (Additional file 3: Fig. S2a and b, respectively). When we separated mice per age group, either 8 or 12 months, a more significant decrease in vascular PDGFRβ signal was detected at the earlier age of 8 months in ADtg mice as compared to matched WT mice (Fig. 2c and Additional file 3: Fig. S2b). We did not notice any sex-related differences in vascular PDGFRβ expression, and significant reductions in PDGFRβ were detected in both female and male ADtg mice versus WT controls (Fig. 2d and Additional file 3: Fig. S2c). Importantly, Pearson’s (*r*) correlation analysis revealed that retinal vascular PDGFRβ deficiency significantly correlated with retinal microvascular degeneration (Fig. 2e). This may suggest that PDGFRβ downregulation is indeed accompanied by capillary degeneration, similar to what is known in other retinal vascular degenerative diseases such as DR [57].

### Vascular Aβ deposition in ADtg mice

Next, we investigated whether Aβ accumulates in retinal blood vessels of these double-transgenic ADtg mice. To achieve this, we first immunostained isolated retinal vasculature with 4G8 to visualize Aβ, together with lectin staining for retinal blood vessels and DAPI nuclei staining (Fig. 3 and Additional file 3: Fig. S3). While 4G8^+^-Aβ signal was absent in perfused WT mice, in perfused ADtg mice, strong Aβ signal was present in retinal blood vessels (Fig. 3a–b). Aβ was found to accumulate in blood vessel walls, vascular cells, or attached to endothelial cells from the lumen side (Fig. 3a–d, g–g′ and Additional file 3: Fig. S3a–b, i–j). Stereological quantification of Aβ confirmed a significant increase in Aβ in ADtg mice when we analyzed all mice together (Fig. 3e; see Additional file 3: Fig. S3c for data normalized per lectin-IR area) and when we separated the animals into 8-month-old and 12-month-old groups (Fig. 3f; see Additional file 3: Fig. S3f for data normalized to lectin-IR area). However, there was no statistically significant difference between the different age groups. Similar to PDGFRβ, both 12-month-old ADtg sexes showed significant increases in vascular 4G8-Aβ burden (Additional file 3: Fig. S3d–e), with males reaching statistical significance probably due to larger sample size. No significant correlation was observed between retinal vascular 4G8-Aβ burden and capillary degeneration (Additional file 3: Fig. S3g) or vascular PDGFRβ expression (Additional file 3: Fig. S3h). Importantly, we revealed that Aβ signal in isolated retinal blood vessels often accumulated in vessel walls and colocalized with lectin (Fig. 3g–g′ and Additional file 3: Fig. S3b). Furthermore, by using 11A50-B10 staining in isolated vasculature, we demonstrated the specific accumulation of Aβ_40_ alloforms in retinal blood vessels of perfused ADtg mice (Additional file 3: Fig. S3i–j). On another occasion, by confocal virtual cross-section, Aβ_40_ deposition in retinal vasculature was detected as encored to Lectin^+^-vessel wall from the lumen side (Additional file 3: Fig. S3j). The localization of Aβ_40_ deposits in retinal blood vessels of ADtg mice is further demonstrated in two 3D-movies (Additional file 1: S1 and Additional file 2: S2 Movies). Finally, we prepared retinal cross-sections from the same mice cohort and validated abundant retinal vascular Aβ accumulation based on double-positive fluorescent staining of thioflavin-S^+^ fibrillar Aβ and 11A50-B10^+^ Aβ_40_ colocalized with CD31^+^ endothelial cells in ADtg mice as compared to WT mice (Fig. 3h–i and Additional file 3: Fig. S3k–n).

### Differential expression of BRB tight junction components and increased retinal NF-κB p65 phosphorylation in ADtg mice

**Fig. 4:**
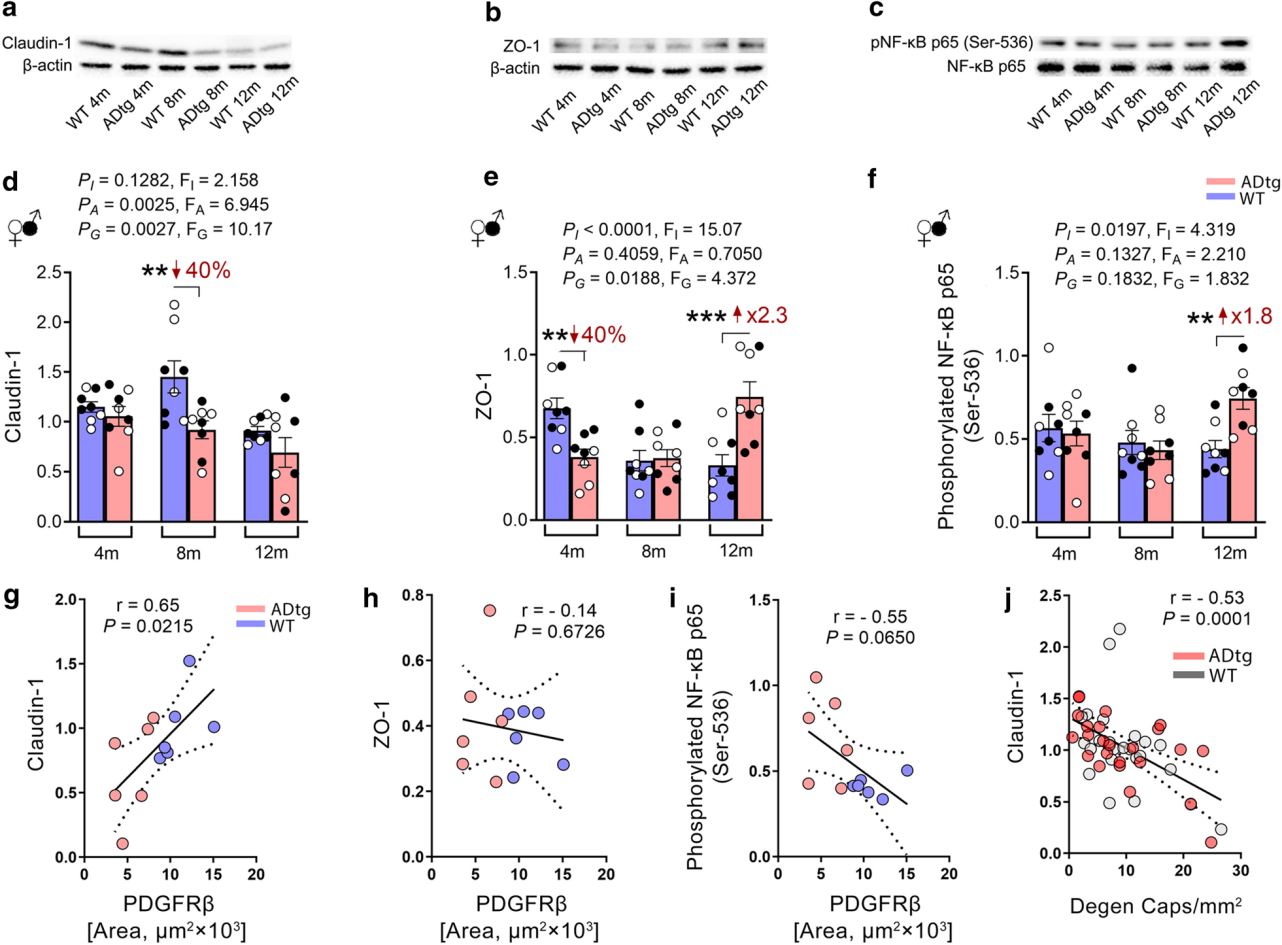
Altered expression of endothelial cell junction molecules and increased NF-κB phosphorylation in the retinas of APP_SWE_/PS1_ΔE9_ (ADtg) mice. **a–c** Representative Tris–glycine gel images showing western blot protein bands of claudin-1, zonula occludin-1 (ZO-1), phosphorylated NF-κB p65 subunit (pNF-κB p65) at serine-536, and total NF-κB p65 subunit as well as β-actin controls from retinal lysates of ADtg mice and age- and sex-matched wild type (WT) littermates (n = 8 for each age and genotype). **d–f** Densitometric analyses of western blot protein bands of **d** claudin-1, **e** ZO-1 and **f** pNF-κB p65, normalized by **d–e** β-actin control or **f** total NF-κB p65 in the same mice cohort. **g–i** Pearson’s coefficient (*r*) correlation between retinal PDGFRβ immunoreactivity and the densitometric analysis of western blot protein bands of **g** claudin-1, **h** ZO-1, or **i** pNF-κB p65 in a subset of this mice cohort (n = 12). **j** Pearson’s coefficient (r) correlation between degenerated retinal capillary count and the densitometric analysis of western blot protein bands of claudin-1. Data from individual mice (circles) as well as group means ± SEMs are shown. Black-filled circles represent males and clear circles represent females. Fold or percentage changes are shown in red. **p < 0.01, ***p < 0.001, by two-way ANOVA with Tukey’s post hoc multiple comparison test. *P* and *F* values of two-way ANOVA refer to comparisons of age groups (*P*_A_, *F*_A_), ADtg versus WT genotype groups (*P*_G_, *F*_G_), interactions (*P*_I_, *F*_I_)

The discovery of exacerbated PDGFRβ loss and capillary degeneration together with vascular Aβ deposition in the retinas of ADtg mice raises the question of whether these small-vessel pathologies are related to cell-to-cell molecular BRB junction disruptions. To address this, we extracted protein homogenates from retinas obtained from ADtg mice and WT controls at 4, 8, and 12 months of age. We measured the protein levels of key tight junction components of the blood-tissue barrier, claudin-1 and zonula occludens–1 (ZO-1) [35, 52, 95, 131], by western blot (WB) analyses (Fig. 4a–b). We also assessed phosphorylation of NF-κB p65 at Ser-536 for potential activation of the relevant inflammatory cascades (Fig. 4c). Densitometric analysis of WB images showed that as compared to matched WT littermates, there were early and significant decreases of retinal claudin-1 and ZO-1 in 8- and 4-month-old ADtg mice, respectively (Fig. 4d–e). Unexpectedly, a significant increase in retinal ZO-1 was detected in 12-month-old ADtg mice. Further, in the 12-month-old ADtg mice, a substantial increase in retinal NF-κB p65 phosphorylation was observed (Fig. 4f), suggesting a heightened inflammatory response. Next, we evaluated if aging played a role in the alteration of these vascular junction molecules. In WT mice, we noticed a 37% decrease in retinal claudin-1 at 12 months of age when compared to animals 8 months old (Additional file 3: Fig. S4a). As for retinal ZO-1, we found 47–51% decreased levels in WT mice at both 8 and 12 months of age as compared with the 4-month-old WT group (Additional file 3: Fig. S4b). The opposite patterns were detected in ADtg mice, where retinal ZO-1 was increased at 12 months (Additional file 3: Fig. S4b). Phosphorylation of NF-κB p65 did not appear to be affected by age (Additional file 3: Fig. S4c). When we regrouped mice by sex and genotype, no significant differences in any of the three molecules were found (Additional file 3: Fig. S4d–f). Pearson’s (*r*) correlation analyses revealed a moderate, positive correlation between retinal claudin-1 levels and retinal capillary PDGFRβ expression (Fig. 4g), but no correlation with retinal ZO-1 (Fig. 4h), suggesting claudin-1 is associated with retinal capillary degeneration in this mouse model. A trend of correlation was also found between NF-κB p65 phosphorylation levels and vascular PDGFRβ expression (Fig. 4i), suggesting that activation of inflammation could play a role in pericyte loss in the ADtg mice retina. Finally, we detected a highly significant inverse correlation between retinal claudin-1 levels and the extent of retinal capillary degeneration (Fig. 4j; see extended correlation analyses in Additional file 3: Fig. S4g–k).

### Retinal microvascular leakage in ADtg mice

**Fig. 5:**
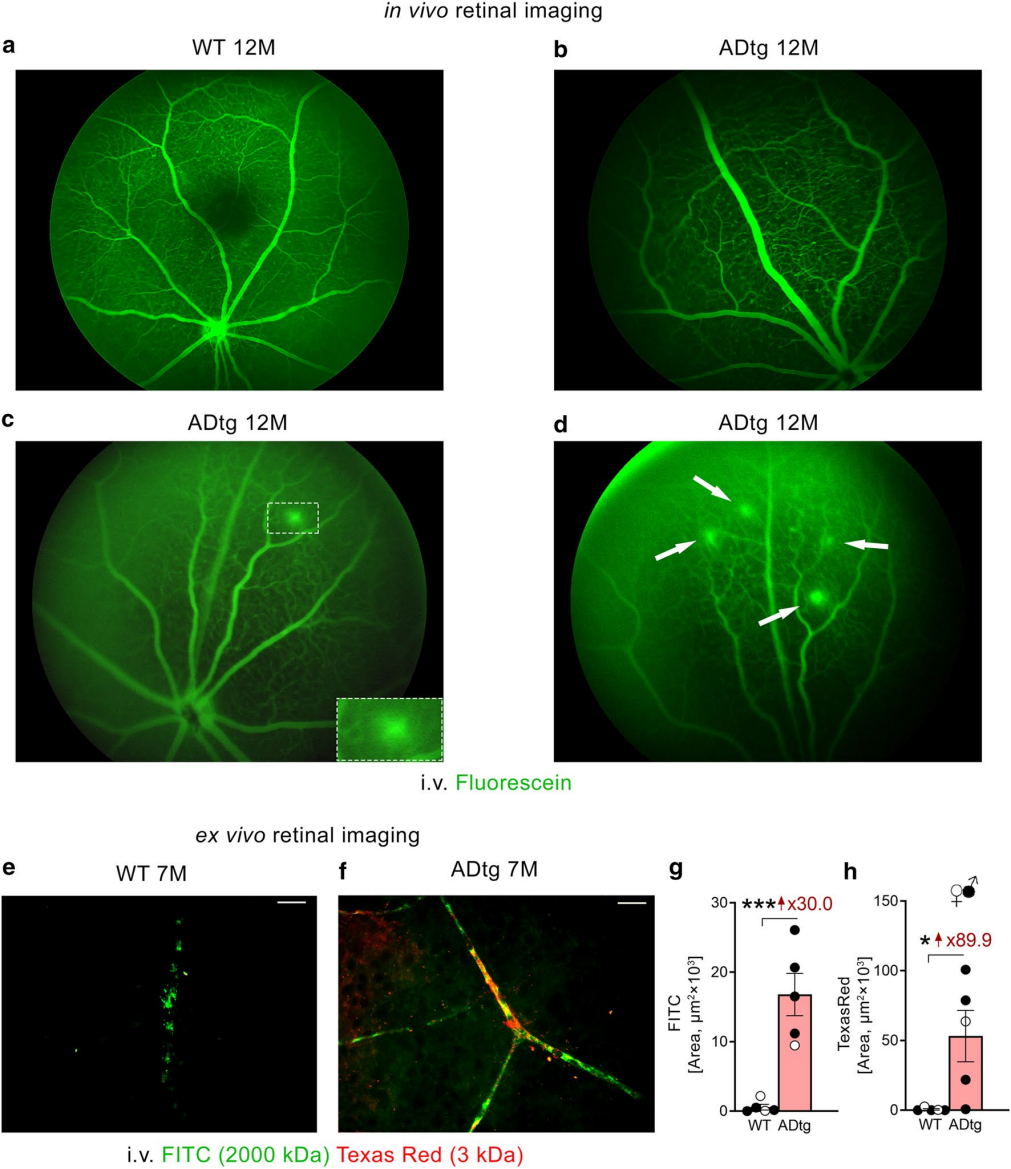
Retinal microvascular leakage in APP_SWE_/PS1_ΔE9_ (ADtg) mice. **a–d** Representative images of noninvasive retinal microvascular imaging after intraperitoneal fluorescein injection in 12-month-old **a** wild type (WT) and **b–d** ADtg mice. Note: images in **b** showing intact retinal microvasculature and in **c–d** showing leaked retinal microvasculature. **e–f** Representative images of retinal flat-mount obtained from 7-month-old WT and ADtg mice (cohort average age is 6 month) that received intravenous tail injections of FITC-dextran and Texas Red-dextran. **g–h** Quantitative analysis of the FITC or Texas Red-stained area in each microscopic field of retinal flat-mounts from WT (n = 5) or ADtg (n = 5) mice. Black-filled circles represent males and clear circles represent females. *p < 0.05, ***p < 0.001, by an unpaired 2-tailed Student *t*-test. Fold changes are shown in red

So far, we have demonstrated increased degenerated capillaries, vascular amyloidosis, and altered cell–cell junctions in the retina of ADtg mice, in comparison with their age- and sex-matched WT littermates. The next question is whether such changes lead to impaired BRB permeability and retinal vascular leakage in ADtg mice. To determine this, we first injected fluorescein (~ 0.3 kD) intraperitoneally in ADtg and WT mice. Using the noninvasive Micron-III retinal imaging microscope [77–79], we detected no fluorescein leakage in any of the WT mice at 8, 12 or 16 months of age (Fig. 5a; a representative image from a 12-month-old WT mouse shown). However, we were able to identify fluorescein leakage in one out of three 12-month-old ADtg mice (Fig. 5c–d, dotted lines or arrows indicating spots of leakage). A representative image from a 12-month-old ADtg mouse with no clear retinal vascular leakage phenotype is shown in Fig. 5b; representative images from 8- and 16-month-old ADtg mice are included in Additional file 3: Fig. S5a–b. We further investigated BRB permeability in AD by co-injecting two compounds with larger predefined molecular weights, FITC-dextran (2000 kD) and Texas Red-dextran (3 kD), into the tail veins of ADtg mice and age- and sex-matched WT mice. Thirty minutes following i.v. injection, the perfused mice were sacrificed, eyes were extracted and whole retinas were isolated. Ex vivo retinal flat-mount imaging revealed intense FITC and Texas Red signals in retinal blood vessel walls and retinal parenchyma of ADtg mice relative to the WT littermates (Fig. 5e–f). Our stereological quantification confirmed substantial 30- to 90-fold increases in both retinal FITC and Texas Red signals, respectively, in ADtg mice (Fig. 5g–h).

## Discussion

In the present study, we provide the first evidence for age-dependent retinal capillary degeneration that strongly associated with PDGFRβ deficiency and co-occurred with Aβ deposits in retinal blood vessels of the double-transgenic APP_SWE_/PS1_∆E9_ mouse model. Retinal vascular changes in murine AD models were apparent at younger ages of 4 and 8 months and tightly correlated with severity of retinal pericyte biomarker (PDGFRβ) deficiency, suggesting pericyte loss occurs early in the retina of amyloidosis-derived AD models. The prominent accumulation of vascular Aβ in the retina of this AD-model mice agrees with earlier findings in a different mouse model of AD (Tg2576) [88] and with our evidence of vascular amyloidosis in postmortem retinas of MCI and AD patients [78, 81, 115]. Further assessment of tight junction-associated proteins from neural retinal lysates showed alterations in claudin-1, ZO-1, and inflammatory-related NF-κB p65 phosphorylation, which all point to an impaired BRB in Alzheimer’s-like retina. Finally, peripheral injection of molecules of increased sizes, fluorescein (~ 0.3 kD), Texas Red-dextran (3 kD) and FITC-dextran (2000 kD), revealed in vivo and ex vivo microvascular leakage in ADtg mouse retina. Taken together, our results broaden the current understanding of retinal microvascular degeneration and BRB integrity in AD murine models, providing new potential targets for AD therapy and encouraging the use of noninvasive retinal vascular imaging for AD diagnosis.

In the Alzheimer’s brain, endothelial cell death [62, 68], tight junction damage [11, 17, 75, 91], and pericyte and vascular smooth muscle cell (vSMC) degeneration [26, 70, 109, 111] were determined to lead to the disruption of NVUs and breakdown of the BBB. Our recent study investigated these pathologies in the retina, revealing early and substantial pericyte apoptotic cell death and PDGFRβ deficiency in postmortem retinas obtained from MCI and AD patients [115]. In agreement, our results here demonstrated capillary degeneration along with PDGFRβ downregulation, overall indicating a microvascular damage in the AD retina. Since previous studies have extensively described neurodegeneration in the AD retina [74, 99, 142], the findings of our current study support the coexistence of neuronal and vascular damage in the AD retina. Another result to note here is the significant correlation between PDGFRβ and degenerated capillaries. In fact, pericyte loss and the decreased ratio to retinal endothelial cells (1:4) is believed to foretell retinal capillary degeneration in DR [42, 105]. DR is a typical retinal vascular degenerative disease where early pathological signatures involving retinal pericyte loss and capillary degeneration are thought to lead to microaneurysm, progressive microvascular leakage, abnormal growth of blood vessels, neurodegeneration, and eventually vision loss [12, 27, 57]. However, an increasing number of studies have provided evidence to support an earlier neuronal dysfunction in the DR retina, potentially indicative of ganglion cell function loss, which predicted subsequent local micro-vasculopathy and macular edema [2, 122]. According to these and other studies, it is suggested that DR primarily affects retinal neuronal function and neurodegeneration, which in turn induce vascular complications [2, 117, 122, 123]. Similarly, our results revealing early retinal micro-vasculopathy and tight-junction molecular changes in AD may indicate a retina-specific neurovascular consequence of age-dependent disturbances between interactions of multiple cell types, such as neuronal, vascular, and perivascular cells. However, the specific mediators of such crosstalk between vascular abnormalities and neurodegeneration have not yet been identified in the AD retina. In any case, our results show that retinal microvascular degenerative pathologies are extensively implicated in the AD retina. Along with previous data showing neuronal function and neurodegeneration in ADtg mouse retina [58, 94], this study contributes to the current understanding of Alzheimer’s-related retinal NVU manifestation. Future studies should aim to investigate the potential impact of neurodegeneration on vasculopathy and further identify specific mediators linking vascular abnormalities and neurodegeneration in the AD retina.

Although retinal Aβ deposition in the APP_SWE_/PS1_∆E9_ (ADtg) mouse model has been extensively described by us and others [24, 46, 49, 55, 77, 93, 97, 102, 140], the only clear demonstration of vascular Aβ accumulation in the murine model so far was based on the Tg2576 mice model in 2009 [88]. In the same year, Dutescu et al. [39] published the first report of amyloid precursor protein overexpression in the ganglion cell layers and inner nuclear layers in retinas of APP_SWE_/PS1_∆E9_ mice. Using a curcumin-based method and confirming ex vivo with various epitope-specific anti-Aβ antibodies, our group was the first to image Aβ plaques in the retina of the same model in vivo [77–79], which was recently corroborated by Sidiqi et al. [116]. In the current study, by using a modified retinal microvascular isolation technique and immunofluorescence staining also in retinal cross sections, we have now provided the first illustration and quantification of retinal vascular Aβ accumulation in the double-transgenic APP_SWE_/PS1_∆E9_ mouse model. The patterns of retinal vascular Aβ deposits in this study—in blood vessel walls, inside vascular and perivascular cells and attached to endothelial cells from the lumen side—appears similar to the CAA patterns reported in AD brains [14, 36, 76, 104]. Future studies should investigate the specific distribution of retinal vascular Aβ deposition across vascular compartments and layers in this animal model. Interestingly, no significant correlation was found here between retinal capillary degeneration and vascular Aβ burden. It is possible that the extent of retinal capillary degeneration may not be directly connected to levels of vascular Aβ but rather is affected by loss of pericytes and neurons, PDGFRβ deficiency, toxicity of abluminal retinal Aβ deposits, detrimental inflammatory reaction, or other indirect consequences of the disease. Yet, since cerebral and retinal Aβ plaques were reported to accumulate in the APP_SWE_/PS1_ΔE9_ transgenic mouse before 6–7 months of age [47, 63, 77, 90], retinal vascular degeneration may be driven by this early Aβ pathology. It is important to note that our correlation analysis was limited by a smaller sample size; future studies should explore these correlations in a larger cohort as well as determine how early vascular degeneration is initiated in the AD retina.

Endothelial cell junctions are indispensable parts of the BBB and inner BRB (iBRB) in maintaining cerebral and retinal homeostasis [35, 84, 137]. Particularly, CNS tissues possess an enriched expression of tight junctions due to the need for maintenance of the blood barriers [84]. The claudins form the backbone of tight junction stands and are pivotal in their transmembrane section, while the zonula occludens are located in the cytoplasm and connect the transmembrane parts of tight junctions to the cytoskeletons [84]. Previously, significant downregulation of ZO-1, claudin-5, and occludin were described in both postmortem human cerebral capillaries with CAA and in 5xFAD transgenic mice [21, 22, 100]. Here, we found decreased levels of claudin-1 in ADtg mice at 4 and 8 months of age but increased levels of ZO-1 at 12 months of age compared to control mice. Our results revealed dysregulation of endothelial tight junctions in the iBRB of ADtg mice. Specifically, downregulation of claudin-1 can indicate damage to the transmembrane part of the retinal endothelial tight junction in mice as young as 8 months old. However, increased levels of ZO-1 in 12-month-old ADtg mice possibly represent a compensatory mechanism in the endothelial cytoplasm in response to tight junction alteration. Importantly, significant correlations were found between retinal claudin-1 and both retinal PDGFRβ and capillary degeneration. These results imply that claudin-1 may be the best biomarker for BRB breakdown in the AD retina. To date, this is the first evaluation of tight junction molecules in the double-transgenic APP_SWE_/PS1_∆E9_ mouse model. Future studies should further investigate other important components of the BRB in the AD retina, such as occludin, claudin-5, desmosomes, and gap and adherens junctions, as well as outer BRB (oBRB) integrity.

The NF-κB protein complex plays a pivotal role in regulating host immune and inflammatory responses by regulating transcription of cytokines and other immune mediators [89]. Of the five subunits in this complex, p65 is the best characterized subunit and is crucial in activating cytokine production [51]. Phosphorylation of NF-κB p65 is a prerequisite for its translocation into the nucleus and binding to target genes [25, 130]. In the current study, we uncovered an increase in phosphorylation of NF-κB p65 in retinas from 12-month-old ADtg mice compared to healthy WT controls. This may indicate an upregulated inflammatory response in the diseased retina. It is important to note that an augmented NF-κB response is implicated in retinal degeneration [136, 141], retinal inflammation [113, 114, 139], as well as in the AD brain [66, 67]. Here, our results provide the first evidence of upregulated NF-κB activity in the retina of the double-transgenic APP_SWE_/PS1_∆E9_ mouse model. Importantly, a previous study utilizing bovine retinal endothelial cells and rat retinas found that the tumor necrosis factor-α-activated NF-κB pathway led to downregulation of tight junction molecules and increased retinal endothelial permeability [9]. Thus, our results of upregulated retinal NF-κB phosphorylation may underlie the molecular mechanisms involved in increased retinal microvascular permeability and iBRB breakdown. We also observed a near significant correlation (*P* = 0.0650, Pearson’s *r* = − 0.55) between increased NF-κB phosphorylation and retinal vascular PDGFRβ deficiency, suggesting a possible relationship between NF-κB activity and pericyte loss in the AD retina.

In the present study, we revealed substantial live fluorescein leakage in the 12-month-old ADtg mice, but not in any of the 8-month or 16-month-old ADtg mice or control animals. We postulate that ADtg mice may present retinal microvascular damage and leakage, specifically related to fluorescein’s molecular structure and size, around 12 months of age. Further, these disruptions may transform into other type of BRB abnormality when animals become older. A limitation here is that we studied only 8 mice in vivo in this cohort. Interestingly, a recent study based on the C57BL/6 mouse revealed decreased plasma protein transport activity through the BBB in the aged brain, driven by transport shifting from ligand-specific receptor-mediated to non-specific caveolar transcytosis [138]. Future studies should aim to explore such ligand-specific transport versus non-specific transports in the BRB and investigate if these alterations exist in the AD human retina.

Importantly, our examination of flat-mount retinas 30 min following intravenous injections of FITC-dextran (2000 kD) and Texas Red-dextran (3 kD) demonstrated dramatic increases of permeability signals in retinal microvascular walls in ADtg mice compared to WT controls at 6–7 months of age, both for the high molecular weight and the low molecular weight compounds. In comparison, our previous study showed that Texas Red-dextran, but not FITC-dextran, was upregulated in the cerebral vasculature of the same double-transgenic ADtg mice compared to WT controls [82]. Therefore, our data here indicate that the retina in this model may be more susceptible to AD-induced microvascular leakage than the brain.

The APP_SWE_/PS1_ΔE9_ transgenic mice are reported to develop cerebral Aβ deposits by the age of 5–6 months and CAA at 6 months, with abundant plaques in the hippocampus and cortex by 9 months, which continue to build up with age [47, 63, 90]. This mouse model has been well-characterized for behavioral deficits across various cognitive domains, although the time of onset and degree of impairment depended on the specific behavioral tests applied [64]. Typically, spatial memory and learning performance as measured by Morris water maze or Barnes maze is considered normal at 7 months of age and comparable to the non-transgenic mice. The hippocampal-based memory and learning functions are substantially impaired by 12 months [83, 134]. Contextual memory, however, may be impaired as early as 6 months of age, as shown by freezing behavior in fear-conditioning tests [72]. Our current data reveals early changes in the ADtg mouse retina between 4 and 8 months of age, including levels of ZO-1 expression, increased capillary degeneration, PDGFRβ deficiency, as well as vascular amyloidosis and leakage. These findings suggest that retinal vascular damage in ADtg mice may precede cognitive deficits. Importantly, a recent investigation in the cerebral cortex of this mice model demonstrated early pathological changes to capillaries at 4–5 months of age, prior to the appearance of CAA and cognitive impairment [71]. Here, the lack of cognitive data or assessment of retinal vascular Aβ and vascular PDGFRβ in mice younger than 4 months limits our ability to determine how early vascular pathology occurs in the retina and its relationship to cognitive deficits. Future studies should explore if any of the biomarkers tested in this study manifest before cerebral pathology and cognitive impairment in this mouse model.

In summary, our study provides a quantitative evaluation of retinal microvascular and iBRB integrity in the double-transgenic APP_SWE_/PS1_∆E9_ mouse model. We identified early and progressive degeneration of retinal capillaries, PDGFRβ loss, retinal microvascular Aβ accumulation, disrupted tight junctions, induced NF-κB inflammatory response, and retinal microvascular leakage. These results have extended our understanding of microvascular damage in the AD retina and have provided multiple new candidate retinal biomarkers. Our study provides further incentive to examine retinal capillary degeneration via OCTA and BRB leakage by fluorescein fundus imaging. Future developments allowing for retinal PDGFRβ or pericyte imaging by application of techniques such as adaptive optics and high-resolution retinal vascular amyloid imaging should facilitate the detection of more specific vascular damage in AD. Together with the recent developments of OCTA, retinal amyloid imaging, and retinal hyperspectral imaging in AD models, this study introduces novel retinal vascular imaging biomarkers that could be detected via a combined noninvasive retinal imaging approach for AD screening and disease monitoring.

## Supplementary information

**Additional file 1. Supplementary Movie 1**: A 3-D constructed movie of isolated retinal vascular network in an ADtg mouse stained with Aβ (11A50-B10) in magenta, lectin in green and DAPI in blue.

**Additional file 2. Supplementary Movie 2**: A Z-stack constructed movie of isolated retinal vascular network in an ADtg mouse stained with Aβ (11A50-B10) in magenta, lectin in green and DAPI in blue.

**Additional file 3. Supplementary Figures 1–5**.

## Data Availability

The data that support the findings of this study are available from the corresponding author, upon reasonable request.
