# Supplementary material for: Retinal capillary degeneration and blood-retinal barrier disruption in murine models of Alzheimer’s disease
Source: Acta Neuropathol Commun. 2020 Nov 23;8:202. doi: 10.1186/s40478-020-01076-4 (PMC7686701; doi:10.1186/s40478-020-01076-4)
Supplement: Supplementary file 3 — Additional file 3. Supplementary Figures 1–5. [file 40478_2020_1076_MOESM3_ESM.pdf]

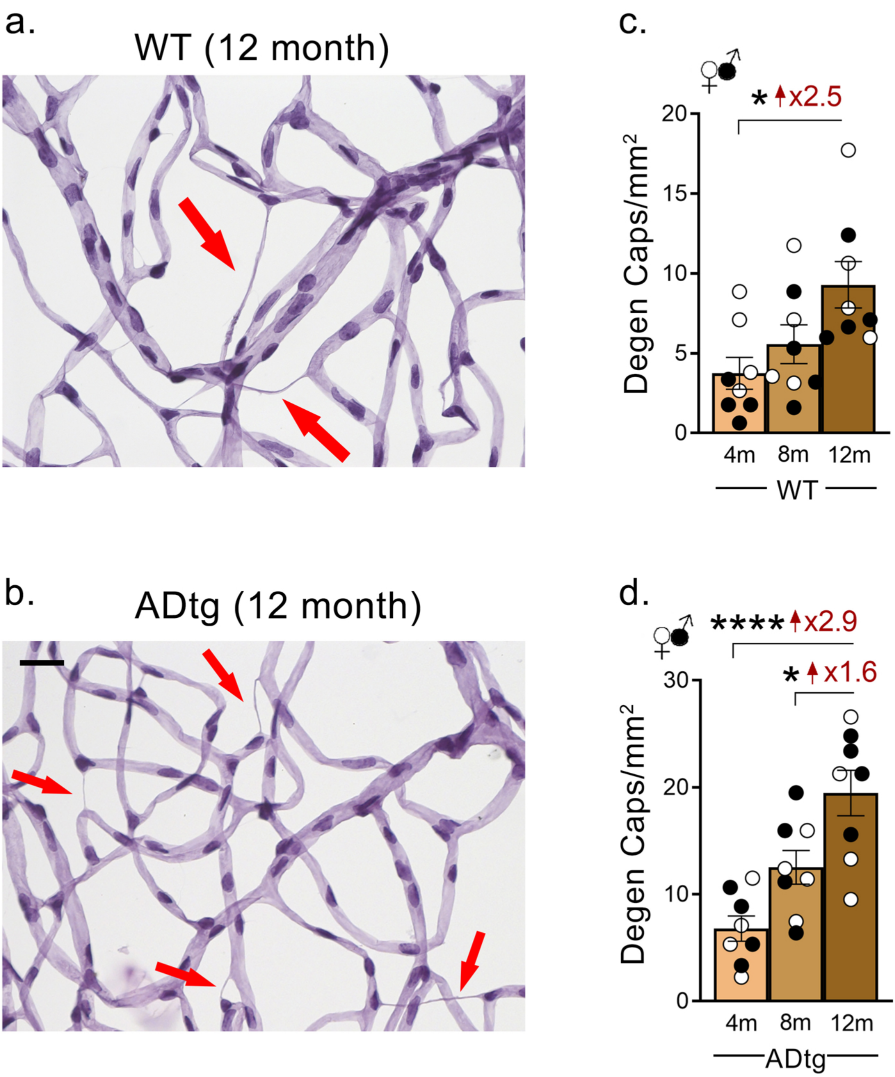

Supplementary figure 1. Addtional representative images and quantifications for figure 1. **a-b**. Representative images of acellular, degenerated retinal capillaries from **a**. a 12-month-old wild type (WT) mouse or **b**. a 12-month-old $APP_{SWE}/PS1_{\Delta E9}$ (ADtg) mouse. Red arrows indicate the degenerated capillaries. Scale bar = 20 μm. **c-d**. Numbers of degenerated retinal capillaries whem mice are stratified by mouse age in **c**. WT or **d**. ADtg mice groups. Data from individual mice (circles) as well as group means ± SEMs are shown. Fold changes are shown in red. Black-filled circles represent males and clear circles represent females. $^{*}p < 0.05$, $^{****}p < 0.0001$, by one-way ANOVA with Tukey's post-hoc multiple comparison test.

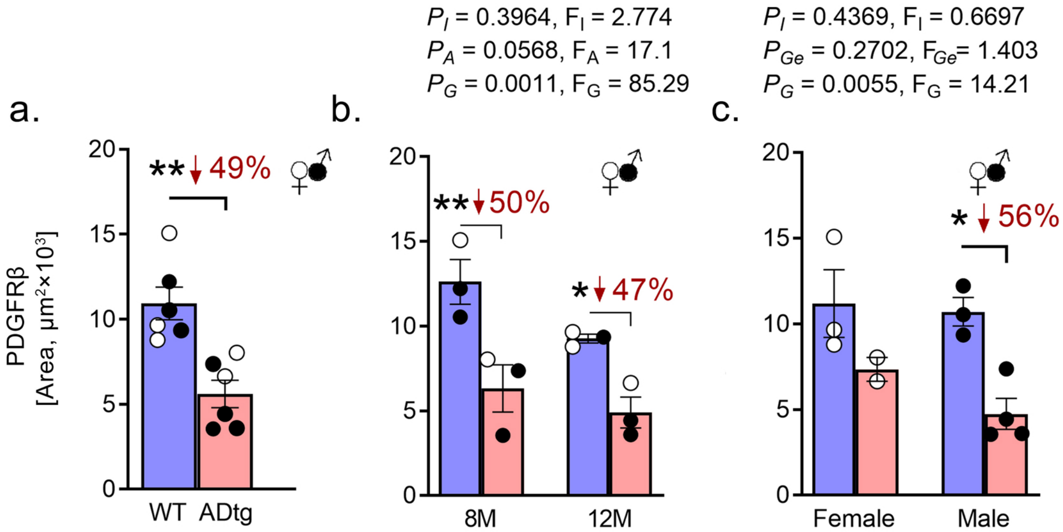

Supplementary figure 2. Raw data of quantification of retinal vascular PDGFRβ. **a**. Quantitative analysis of raw PDGFRβ-immunoreactive (IR) area in each microscopic field of isolated retinal microvasculature from wild type (WT) (n=6) and $APP_{SWE}/PS1_{\Delta E9}$ ADtg (n=6) mice. **b**. Quantification of PDGFRβ-IR in the same mice cohort when mice are stratified by genotypes of WT and ADtg with **b**. age of mice by 8 month and 12 month or **c**. sex of mice. Data from individual mice (circles) as well as from groups are shown with means ± SEMs. Black-filled circles represent males and empty circles represent females; $^{*}p < 0.05$, $^{**}p < 0.01$, by two-way ANOVA with Tukey's post-hoc multiple comparison test. Two group statistical analysis was performed using an unpaired 2-tailed Student t-test. Percentage changes are shown in red.

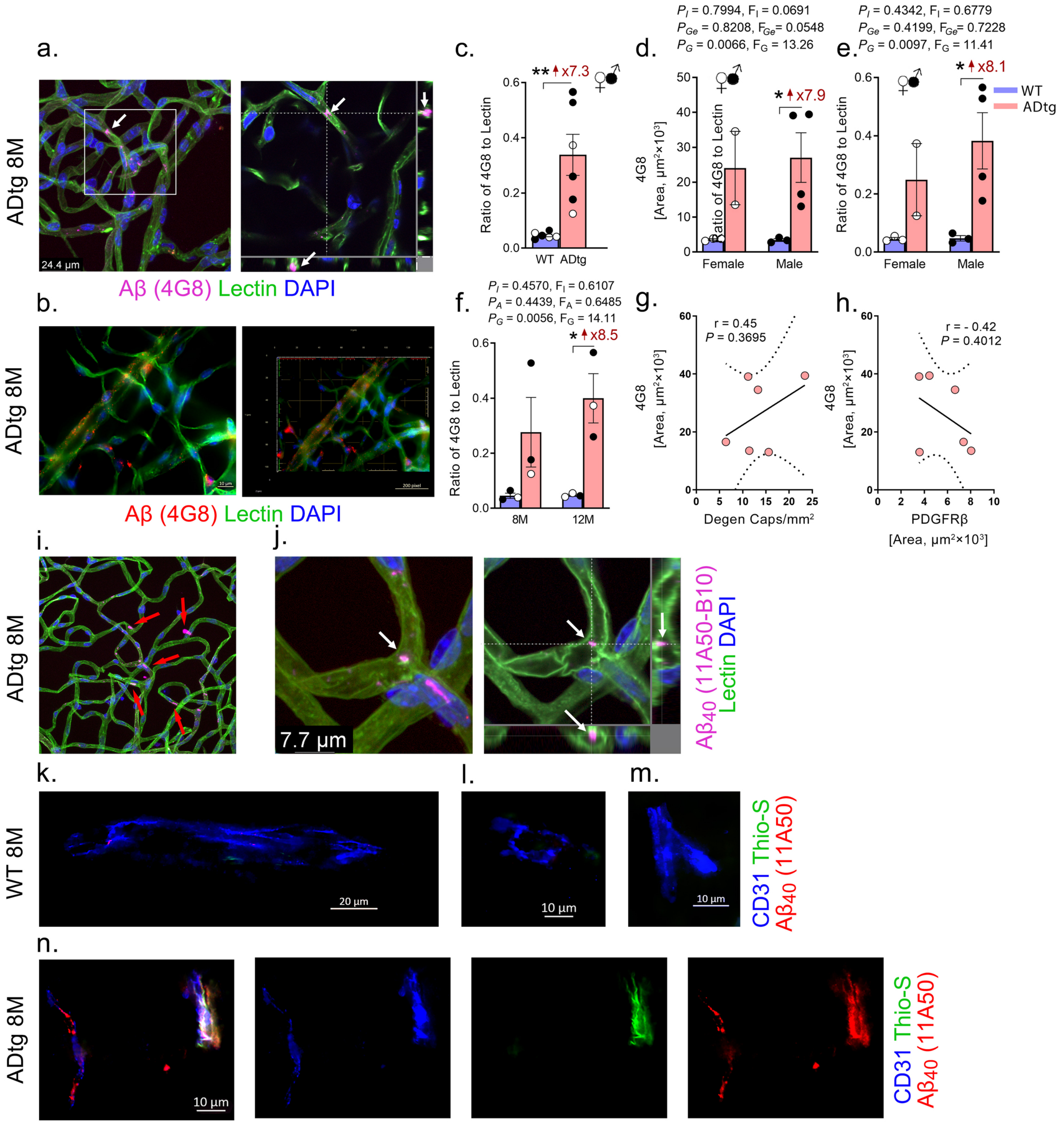

Supplementary figure 3. Supplementary images, quantifications and correlations for figure 3. **a-b**. Representative fluorescence images of isolated retinal microvasculature stained for Aβ (4G8, magenta or red, as indicated under the images), blood vessels (lectin, green), and nuclei (DAPI, blue) in a perfused 8-month-old $APP_{SWE}/PS1_{\Delta E9}$ (ADtg) mice. Arrows indicate vascular Aβ. **c**. Quantitative analysis of the Aβ (4G8)–immunoreactive (IR) area normalized by lectin area in each microscopic field of isolated retinal microvasculature from wild type (WT) (n=6) or ADtg (n=6) mice. **d-e**. Quantitative analysis of the **d**. Aβ (4G8)–IR area or **e**. Aβ (4G8)–IR area normalized by lectin area in each microscopic field of isolated retinal microvasculature from the same cohort separated by different sex of mice. **f**. Quantitative analysis of the Aβ (4G8)–IR area normalized by lectin area separated by different mice age groups (8 months and 12 months) and genotypes (WT and ADtg) in the same cohort. n=3 for each group. **g-h**. Pearson's coefficient (r) correlation between the retinal Aβ (4G8)-IR area against **g**. degenerated capillaries or **h**. PDGFRβ-IR area in ADtg mice (n=6) of this cohort. **i-j**. Representative fluorescence images of isolated retinal microvasculature stained for $A\beta_{40}$ (11A50-B10, magenta), blood vessels (lectin, green), and nuclei (DAPI, blue) in a perfused 8-month-old ADtg mice. Arrows indicate vascular Aβ. **k-n**. Representative fluorescence images of retinal cross-section for thioflavin-S (Thio-S, green), $A\beta_{40}$ (11A50-B10, red) and blood vessels (CD31, blue) in a perfused 8-month-old **k-m**. WT or **n**. ADtg mice. Data from individual mice (circles) as well as from groups are shown with means ± SEMs. Black-filled circles represent males and empty circles represent females; $*p < 0.05$, $**p < 0.01$, by two-way ANOVA with Tukey's post-hoc multiple comparison test. Two group statistical analysis was performed using an unpaired 2-tailed Student t-test. Fold changes are shown in red.

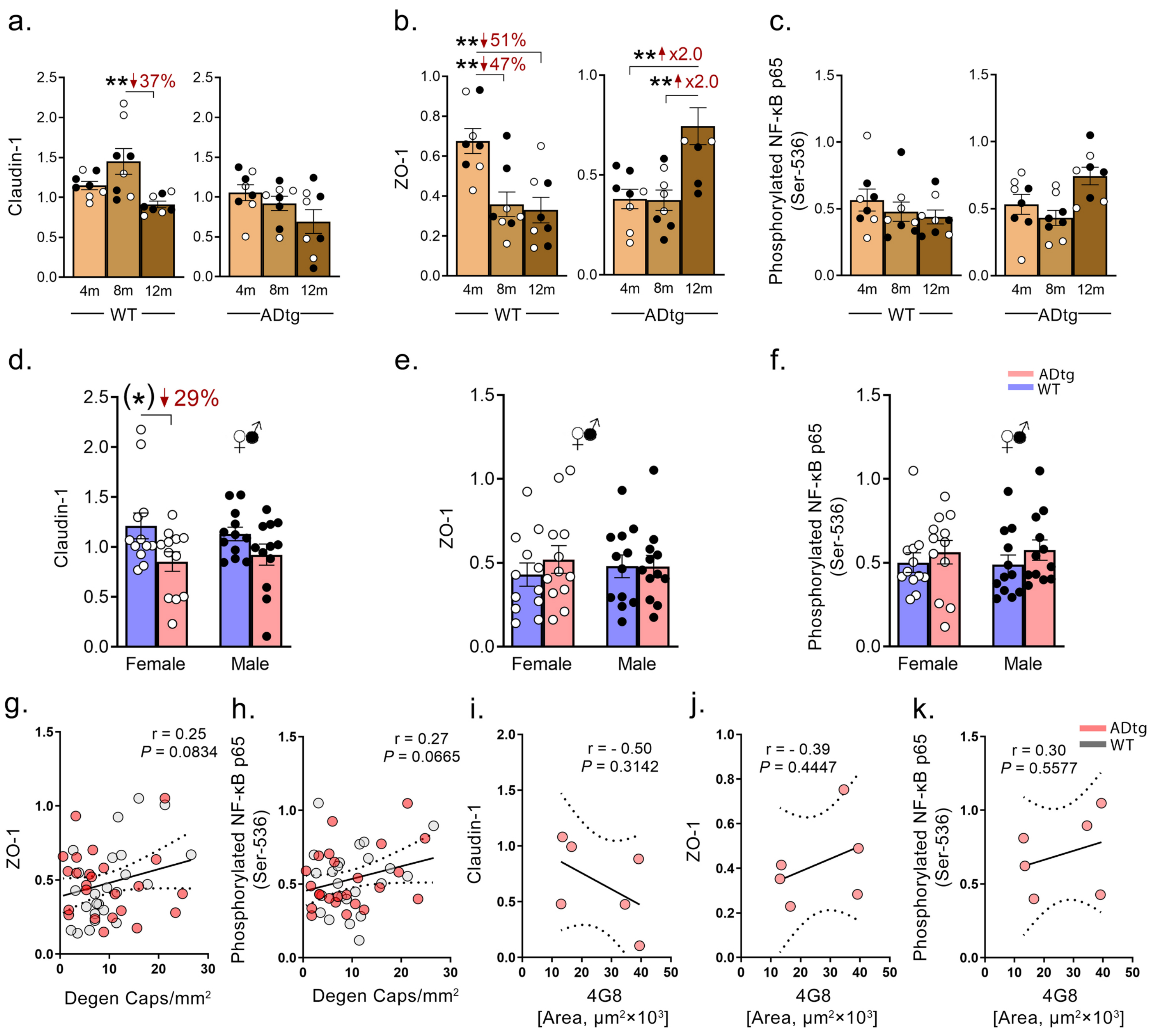

Supplementary figure 4. Additional data for figure 4. **a-c**. Densitometric analysis of western blot protein bands of **a**. claudin-1, **b**. ZO-1, and **c**. pNF-κB p65 with normalization, separated by mice age (4, 8, and 12 months) and genotype (WT and ADtg) in the same mice cohort as Figure 4. n=8 for each group. **d-f**. Densitometric analysis of western blot protein bands of **d**. Claudin-1, **e**. ZO-1 and **f**. pNF-κB p65 in the same mice cohort (n=12 for each group) separated by sex. Data from individual mouse (circles) as well as groups are shown as means ± SEMs. Black circles represent males and clear circles represent females. *$p < 0.05$, **$p < 0.01$, by one-way or two-way ANOVA with Tukey's post-hoc multiple comparison test. Two group statistical analysis was done by an unpaired 2-tailed Student t-test, and is shown in parenthesis. Percentage and fold changes are shown in red. **g-h**. Pearson's coefficient (r) correlation between retinal degenerated capillaries (Degen Caps) and the densitometric analysis of western blot protein bands of **g**. ZO-1, or **h**. pNF-κB p65 in the same mice cohort of figure 4 (n=48). **i-k**. Pearson's coefficient (r) correlation between retinal 4G8-immunoreactive area and the densitometric analysis of western blot protein bands of **i**. claudin-1, **j**. ZO-1, or **k**. pNF-κB p65 in a subset of the $APP_{SWE}/PS1_{\Delta E9}$ (ADtg) mice cohort in figure 4 (n=6).

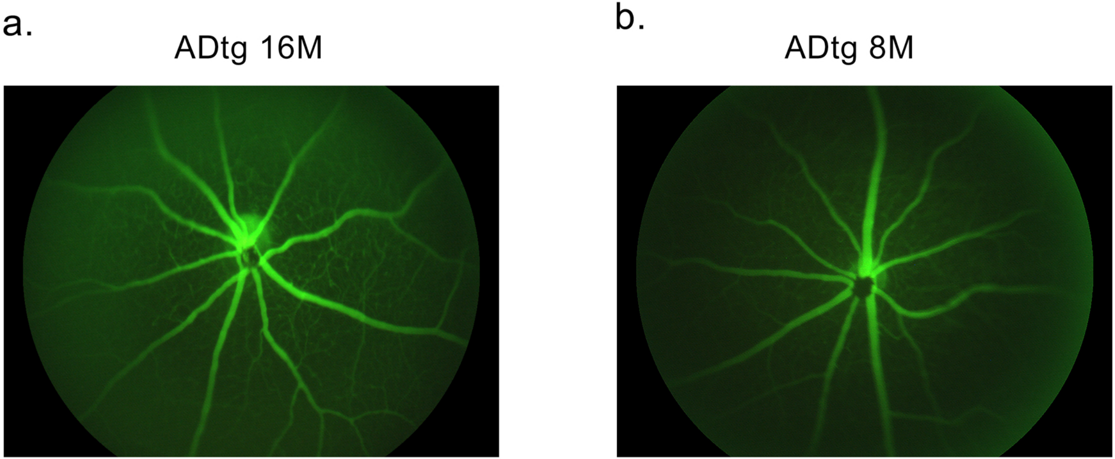

Supplementary figure 5. Additional representative images for figure 5. **a-b**. Representative images of noninvasive retinal microvascular imaging after intraperitoneal fluorescein injection in **a**. 16-month-old $APP_{SWE}/PS1_{\Delta E9}$ (ADtg) and **b**. 8-month-old ADtg mice.
